# Tenascin-C promotes bone regeneration via inflammatory macrophages

**DOI:** 10.1038/s41418-024-01429-9

**Published:** 2025-01-10

**Authors:** Qian Ren, Wenhui Xing, Bo Jiang, Heng Feng, Xuye Hu, Jinlong Suo, Lijun Wang, Weiguo Zou

**Affiliations:** 1 State Key Laboratory of Cell Biology, Shanghai Institute of Biochemistry and Cell Biology, Center for Excellence in Molecular Cell Science, Chinese Academy of Sciences, University of Chinese Academy of Sciences, Shanghai, China; 2 Hainan Medical University, Haikou, Hainan China; 3 Institute of Microsurgery on Extremities, Shanghai Jiao Tong University Affiliated Sixth People’s Hospital, Shanghai, China

**Keywords:** Acute inflammation, Immunological disorders

## Abstract

During the early stage of tissue injury, macrophages play important roles in the activation of stem cells for further regeneration. However, the regulation of macrophages during bone regeneration remains unclear. Here, the extracellular matrix (ECM) tenascin-C (TNC) is found to express in the periosteum and recruit inflammatory macrophages. TNC-deficiency in the periosteum delays bone repair. Transplantation of macrophages derived from injured periosteum is able to rescue the decreased skeletal stem cells and impaired bone regeneration caused by TNC deficiency. The cell communication analysis identifies ITGA7 as a TNC receptor contributing to the recruitment of inflammatory macrophages. TNC expression declines in aged mice and the exogenous delivery of TNC significantly promotes bone regeneration after aging through the recruitment of macrophages. Taken together, this study reveals the regulation of macrophage recruitment and its function in the activation of skeletal stem cells after bone injury, providing a strategy to accelerate bone regeneration by TNC delivery.

## Introduction

Bone injury poses a threat to skeletal homeostasis and even life safety [1, 2]. The revealing of the mechanism of how the orchestration of different kinds of cells during bone regeneration and remodeling happens to support the final recovery of the tissue may enlighten us with a new approach to accelerate the healing process and attenuate the pain of the patients. Generally, bone regeneration is a sequential process, including the formation of hematoma, inflammation, anti-inflammatory, the formation of new bone, and the remodeling of the healing tissue [3]. Dramatic inflammation is an apparent difference between tissue development and regeneration. During inflammation, macrophages are required to respond to the injury signal, clear the cell debris, and secrete key factors including growth factors to communicate with other cell types [4–6]. As key niche cells in the microenvironment of tissue repair, crosstalk between macrophages and other cell types, such as keratinocyte and neuron, plays an important role in injury response [7, 8]. The spatial and temporal regulation of macrophage subtypes including M1 and M2 populations is crucial to efficient tissue repair [9–12]. The presence of M1 macrophages is known to recruit stem cells, promote osteoblast differentiation of stem cells, and promote angiogenesis [13]. Skeletal cells and macrophages closely interact with each other through molecular mediators. Thus, how the osteo-lineage cells contribute to macrophage recruitment and how the macrophages contribute to the function of skeletal stem cells during injury remain to be further investigated.

As the founding member of the tenascin family, the highly conserved ECM, Tenascin-C (TNC), has been found to be exquisitely regulated during development [14]. TNC has epidermal growth factor (EGF)-like domains, fibronectin-type III (FNIII) domain repeats and a fibrinogen-like globe. The multimodular structure enables TNC to interact with a plethora of other molecules [15–17]. Furthermore, the rapid induction of TNC in response to tissue injury in different tissues attracted broad attention. Previous studies have demonstrated that TNC was necessary to normal repair of multiple tissues including skeletal muscle and bone marrow. In the bone marrow, TNC was required to prime the bone marrow microenvironment for hematopoietic regeneration [18]. While in the skeletal muscle, the necroptotic myofibers released TNC and facilitated muscle regeneration through activating EGF receptor (EGFR) signaling [19]. In this study, we investigated whether and how TNC would contribute to bone regeneration.

In the present study, we analyzed the effects of TNC deficiency in periosteal cells labeled by *Prx1 Cre* and found that knockout of *Tnc* in *Prx1*+ cells did not affect the normal development of mice. However, the repair of bone injury was delayed in the absence of TNC. The upregulation of TNC in the early stage of bone repair promoted bone healing through the recruitment of M1 macrophages, which adhered to TNC-expressing cells via Integrin α7 (ITGA7). The recruitment of macrophages to the periosteum increased mSSCs to accelerate the repair of bone injury. TNC expression declined in aged mice and the exogenous delivery of TNC significantly enhanced bone regeneration after aging, providing a strategy to accelerate bone regeneration by TNC delivery.

## Results

### TNC was required in *Prx1*+ cells for efficient bone regeneration

**Fig. 1:**
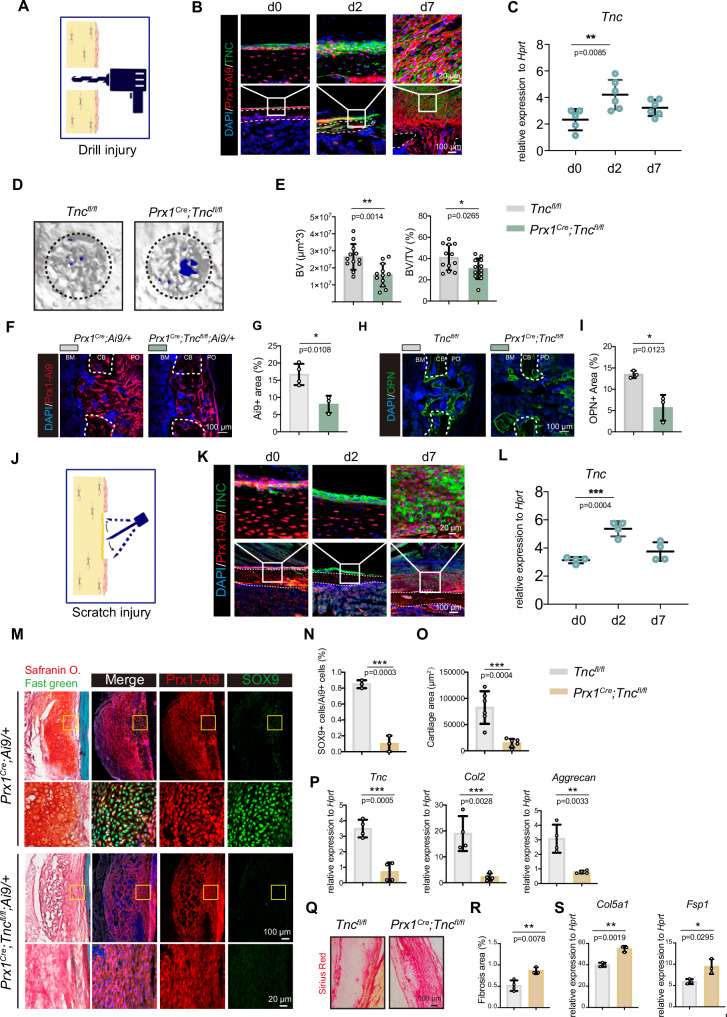
Expression of TNC increased after bone defect injury, and its deficiency in *Prx1*+ cells led to bone repair delay. **A** The schematic diagram of drill injury. **B** Representative immunofluorescence images showing the expression of TNC in the periosteum of wild type (WT) mice at uninjured (d0), 2-day post injury (d2), 7-day post injury (d7). **C** Quantitative RT-PCR detection of the *Tnc* gene expression in periosteal *Prx1*+ cells of *Prx1*^*Cre*^; Ai9/+ mice at d0, d2, d7. *n* = 5 for d0, *n* = 6 for d2, d7. **D** Representative micro-CT images of the drill holes in the femoral bone of *Tnc*^*fl/fl*^ and *Prx1*^*Cre*^;*Tnc*^*fl/fl*^ mice at 14-day post bone defect injury, where the black dashed circle illustrated the regenerated bone in the drill hole. **E** Quantitative measurements of bone volume (BV) and bone volume per tissue volume (BV/TV) of the newly formed bone in the drill holes. *n* = 12. Representative images of *Prx1*^*Cre*^*; Ai9/+* and *Prx1*^*Cre*^; *Tnc*^*fl/fl*^*; Ai9/+* mice (**F**) and the quantification of the Ai9+ area (**G**). *n* = 4 for Ctrl, n = 3 for CKO. The immunofluorescence of the ossification marker osteopontin (OPN) (**H**) and the quantification of the OPN+ area (**I**) in the drill holes of *Tnc*^*fl/fl*^ and *Prx1*^*Cre*^;*Tnc*^*fl/fl*^ mice at 14-day post bone defect injury. *n* = 3. **J** The schematic diagram of periosteum scratch injury. **K** Representative immunofluorescence images showing the expression of TNC in the periosteum of wild type (WT) mice at uninjured (d0), 2-day post injury (d2), 7-day post scratch injury (d7). **L** Quantitative RT-PCR detection of the *Tnc* gene expression in periosteal *Prx1*+ cells of *Prx1*^*Cre*^; Ai9/+ mice at d0, d2, d7 after scratch injury. n = 4. **M** Representative images of periosteum of *Prx1*^*Cre*^*; Ai9/+* and *Prx1*^*Cre*^; *Tnc*^*fl/fl*^*; Ai9/+* mice at d7 of scratch injury. Quantitative results of the chondrocytes marked by Sox9 (**N**) (*n* = 3), the cartilage area (**O**) (*n* = 6) and the expression of the indicated genes of Ai9+ cells at d7 after scratch injury (**P**) (*n* = 4). **Q** Representative images of the Sirius Red staining of the periosteum at d7 after scratch injury. **R** Quantification of the fibrosis area in the periosteum according to the Sirius Red staining. *n* = 4 for Ctrl, *n* = 3 for CKO. **S** Quantitative RT-qPCR examination of the indicated genes in Ai9+ cells at d7 after scratch injury. *n* = 3. The statistical significance of differences was assessed using two-tailed Student’s unpaired *t* test or one-way ANOVA. All bar graphs are presented as the mean ± SD.

To understand the expression kinetics of TNC in the periosteum of the long bone of mice in healthy and injured conditions, we examined TNC expression through immunofluorescence staining and real-time quantitative PCR (RT-qPCR). The defect of cortical bone was realized by drill injury (Fig. 1A). The immunofluorescence staining of TNC indicated that the expression of TNC in the periosteum was rapidly intensified 2 days after the bone defect (Fig. 1B). In line with the immunofluorescence staining, expression of *Tnc* mRNA in the periosteal cells significantly increased at d2 (Fig. 1C). To assess the role of TNC in bone regeneration, we constructed the *Prx1*-dependent TNC conditional knockout (CKO) mice by crossing *Prx1* Cre mice and *Tnc* flox mice, in which the expression of TNC was deleted in the *Prx1*+ lineage cells of the *Prx1*^*Cre*^*;Tnc*^*fl/fl*^ mice. With the combination of *Ai9/Ai9* mice, the *Prx1*+ cells can be labeled by tdTomato in *Prx1*^*Cre*^*;Tnc*^*fl/fl*^*;Ai9/+* and *Prx1*^Cre^*;Ai9/+* mice (Fig. S1A). The immunofluorescence staining presented the expression of TNC in the *Prx1*+ cells in the periosteum while the expression of TNC was absent in the *Prx1*^*Cre*^*;Tnc*^*fl/fl*^*;Ai9/+* mice (Fig. S1B). The RT-qPCR of the periosteal cells of control and *Prx1*^*Cre*^*;Tnc*^*fl/fl*^ mice also validated the usability of the model (Fig. S1C). Staining of TNC in *Prx1*^*Cre*^*;Ai9/+* mice at E15.5 and P1 revealed abundant expression and the extracellular pattern of TNC in *Prx1*+ cells in the periosteum and perichondrium of mice during early development (Fig. S1D), although the embryonic skeletal development was not influenced by the deficiency of TNC (Fig. S1E). Linage-tracing utilizing *Prx1*^*CreERT2*^*;Ai6/+* at d0 and d7 showed the participation of *Prx1*+ cells in the regeneration of bone after bone drill injury and periosteum scratch injury (Fig. S1F).

To test if TNC was required by bone regeneration, we took advantage of the cortical drill hole injury repair model, in which the closure level of the drilled hole determined by μ-CT reflected the efficiency of bone repair and regeneration. The μ-CT analysis showed that the repair of the drill injury in the *Prx1*^*Cre*^*;Tnc*^*fl/fl*^ mice was significantly delayed at 14-day-post injury compared with the littermate *Tnc*^*fl/fl*^ mice (Fig. 1D, E). Histomorphometry analysis revealed that the *Prx1*+ lineage cells in the drilled hole labeled by tdTomato in the *Prx1*^*Cre*^*;Tnc*^*fl/fl*^*;Ai9/+* mice were less than that in the *Prx1*^*Cre*^*;Ai9/+* mice (Fig. 1F, G). Additionally, there was less new-formed bone in the injury site stained by Osteopontin (OPN) in *Tnc*-deficient mice (Fig. 1H, I). In addition, we also performed the periosteum scratch injury model without penetration of the cortical bone (Fig. 1J). The expression kinetics of TNC was similar with that in the drill injury model (Fig. 1K, L). The cartilage formation assessed by the expression of SOX9 was normal in the periosteum of WT mice but impeded in *Prx1*^*Cre*^*;Tnc*^*fl/fl*^ mice (Fig. 1M–O). Consistent with the histological analysis, the expression of chondrogenic markers decreased and the area of fibrosis increased significantly without the presence of TNC (Fig. 1P–S). In conclusion, these data demonstrated that TNC was required for efficient bone repair.

### TNC-deficiency did not impair bone development or differentiation of *Prx1*+ cells

To explore the function of TNC during bone development, we performed a series of morphological analysis. μ-CT scanning of the calvarium at P5 (Fig. S1G) and whole mount skeleton staining at P7 (Figure S1H) showed no significant difference between *Prx1*^*Cre*^*;Tnc*^*fl/fl*^ mice and the littermate control. Deficiency of TNC in *Prx1*+ lineage cells in 4-month-old adult mice did not impair the morphology or body weight of the mice (Fig. S1I, J). Three-point-bending experiment demonstrated normal mechanical property of the bones between the two groups (Fig. S1K). Histological staining and μ-CT quantification analysis of the femur of the 4-month-old mice indicated uninfluenced skeletal histological structure in the conditional knockout mice (Fig. S1L–N).

During the healing process of bone injury, the proliferation and osteogenic differentiation of skeletal stem/progenitor cells and osteoblasts are key factors for successful bone repair. Given that deficiency of TNC in periosteal osteogenic cells delayed the recovery of the injured bone, we interrogated whether the osteogenic and chondrogenic differentiation capability of the periosteal cells were altered by the knockout of TNC. According to the Alizarin Red S, ALP staining and the osteogenic genes quantification of the differentiated cells, the periosteal *Prx1*+ cells isolated from uninjured *Prx1*^*Cre*^*;Tnc*^*fl/fl*^*;Ai9/+* mice and littermate control showed no significant difference in osteogenic differentiation (Fig. S2A–D). CCK-8 assay demonstrated no significant difference in cell proliferation in *Prx1*+ cells with or without the presence of TNC (Fig. S2E). In micromass culture of chondrogenic differentiation assay, TNC-deficiency in *Prx1*+ cells did not influence the chondrogenic differentiation (Fig. S2F, G). Taken together, we found that TNC-deficiency in *Prx1*+ cells did not impair the osteogenic or chondrogenic differentiation of the periosteal *Prx1*+ cells or the normal bone development.

### TNC-deficiency impaired osteogenic and chondrogenic differentiation of periosteal *Prx1*+ cells after bone injury

**Fig. 2:**
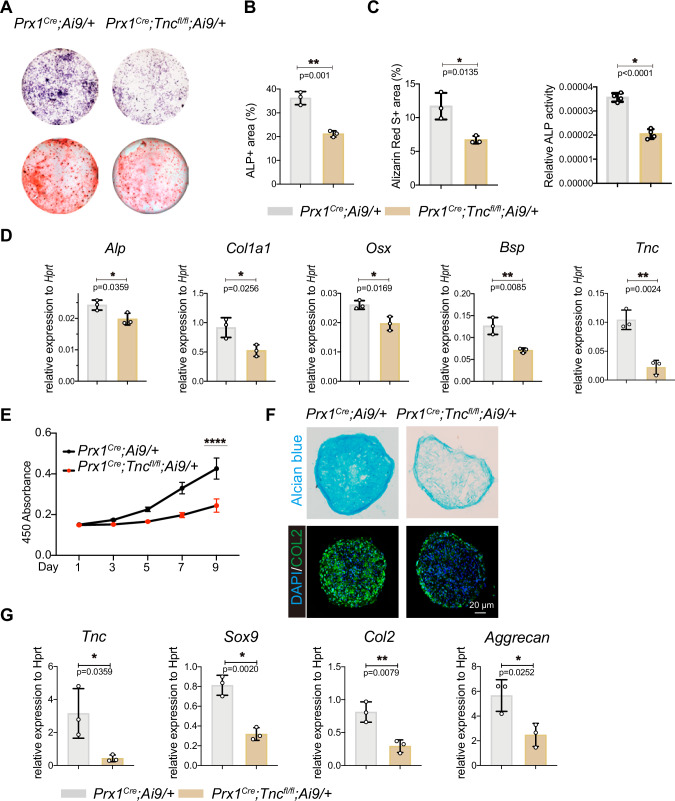
TNC-deficiency impaired osteogenic and chondrogenic differentiation of the periosteal *Prx1*+ cells after bone injury. **A** ALP (upper panel) and Alizarin Red S (lower panel) staining of primary periosteal cells from the indicated mice 2-day post bone drill injury after induction with osteogenic medium for 7 days and 21 days, respectively. Quantification of the percentage of ALP- stained area (**B**) (*n* = 3). Quantification of the percentage of Alizarin Red S area and the relative activity of ALP (**C**) (*n* = 4). **D** Quantitative RT-qPCR detection of osteogenic biomarker genes (*Alp, Col1a1, Osx, Bsp*) and *Tnc* in the osteogenic differentiated cells. *n* = 3. **E** Quantification of cell proliferation of the sorted tdTomato+ periosteal cells from the indicated mice 2-day post bone drill injury in CCK-8 assay. *n* = 6. Representative images of the Alcian blue staining and Col2 staining (**F**) and the expression of the indicated genes of the chondrogenic differentiated periosteal cells after scratch injury (**G**). *n* = 3. The statistical significance of differences was assessed using two-tailed Student’s unpaired t test. All bar graphs are presented as the mean ± SD.

To verify if the alteration of the periosteal cell property is injury-dependent, we isolated the *Prx1*+ periosteal cells 2 days after injury and then conducted the cell behavior examination. ALP, Alizarin Red S staining, and enzyme activity examination showed attenuated osteogenic differentiation of the periosteal cells after TNC deletion (Fig. 2A–C). The expression of osteogenic markers was significantly repressed in cells from *Prx1*^*Cre*^*;Tnc*^*fl/fl*^*;Ai9/+* mice (Fig. 2D). CCK-8 assay presented reduced cell proliferation of TNC-deficient cell (Fig. 2E). Chondrogenic differentiation was attenuated without the presence of TNC (Fig. 2F, G). Taken together, we found that TNC in periosteal *Prx1*+ cells was required for the normal osteogenic and chondrogenic differentiation after bone defect injury.

### TNC mediated the recruitment of macrophages during bone repair

**Fig. 3:**
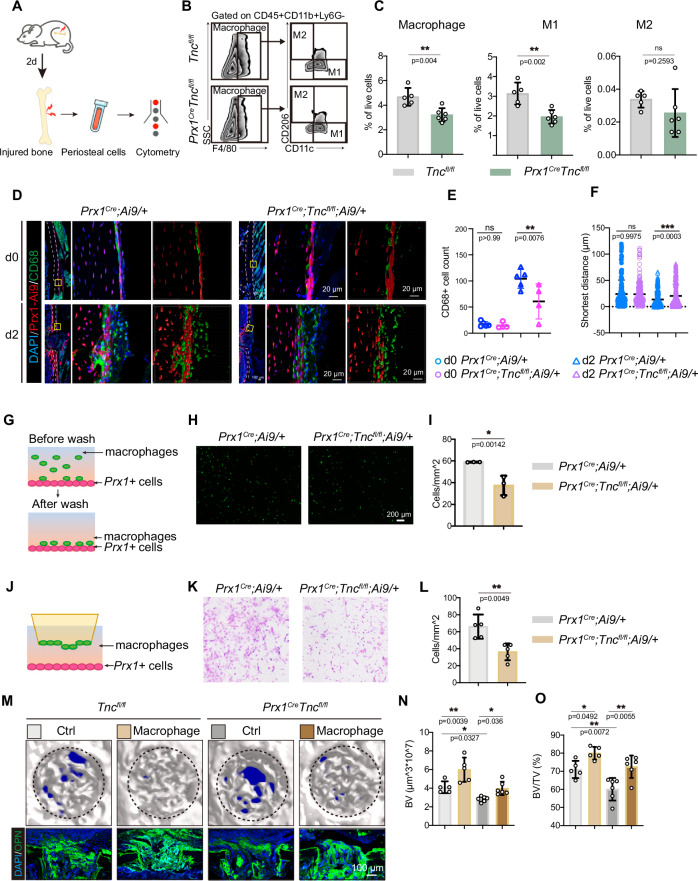
Decreased macrophage recruitment after bone injury in *Prx1*^*Cre*^;*Tnc*^*fl/fl*^ mice. **A** Scheme of the experiment: 2 days after the femoral bone of *Tnc*^*fl/fl*^ and *Prx1*^*Cre*^;*Tnc*^*fl/fl*^ mice were drill injured, the percentage of macrophages in the periosteal cells were analyzed by flow cytometry. **B** Flow cytometry analysis of the periosteal macrophages at 2-day post injury. **C** Quantification of the percentage of total macrophages and M1, M2 macrophages in the periosteal cells at 2-day post injury. *n* = 5 for Ctrl, *n* = 6 for CKO. **D** Representative immunofluorescence images and their corresponding images reconstructed by imaris of the periosteum in *Prx1*^*Cre*^*; Ai9/+* and *Prx1*^*Cre*^*; Tnc*^*fl/fl*^*; Ai9/+* mice at d0 and d2, where tdTomato and CD68 signal marking the periosteal *Prx1*+ cells and macrophages, respectively. Quantification of the CD68+ cells (**E**) and the shortest distance between tdTomato+ cells and CD68+ cells (**F**) in the periosteum at indicated mice and time points. *n* = 4 or 5. **G** Experiment scheme of the adhesion assay: confluent primary periosteal *Prx1*+ cells from the periosteum of *Prx1*^*Cre*^*;Ai9/+* and *Prx1*^*Cre*^*;Tnc*^*fl/fl*^ *;Ai9/+* mice at d2 were seeded in the culture dish and the macrophages from the periosteum of WT mice at d2 stained by Calcein-AM were added in the dish for 30 min for cell adhesion before the un-adhered cells were washed. Representative images (**H**) and quantification (**I**) of the adhered macrophages in the adhesion assay. *n* = 3. **J** Experiment scheme of the trans-well assay: the periosteal *Prx1*+ cells from the periosteum of *Prx1*^*Cre*^*;Ai9/+* and *Prx1*^*Cre*^*;Tnc*^*fl/fl*^ *;Ai9/+* mice at d2 were seeded in the lower chamber to induce the migration of the macrophages from the periosteum of WT mice at d2 seeded in the upper chamber. Representative images (**K**) and quantification (**L**) of the migrated macrophages (stained by crystal violet). *n* = 5. **M** Representative micro-CT images and immunofluorescence images of the drill holes with transplantation of macrophages from the periosteum of WT mice at d2 in the femoral bone of *Tnc*^*fl/fl*^ and *Prx1*^*Cre*^;*Tnc*^*fl/fl*^ mice at 14-day post bone defect injury. Quantitative measurements of bone volume (BV) (**N**) and bone volume per tissue volume (BV/TV) (**O**) of the newly formed bone in the drill holes of the indicated mice with macrophages transplantation. *n* = 5 or 6. The statistical significance of differences was assessed using two-tailed Student’s unpaired t test or two-way ANOVA. All bar graphs are presented as the mean ± SD.

Drastic inflammation response is one of the most obvious differences between bone morphogenesis during development and bone regeneration during tissue repair. The destruction of blood-supply system and the release of injury-related signals in the early stage of tissue repair bring a large number of immune cells to the injury site to initiate the regeneration process. Since the deficiency of TNC in *Prx1*+ cells did not impair the skeletal development of the mice under homeostasis, we wondered if it was the altered immune microenvironment in the early stage of bone repair after the deletion of *Tnc* that affected the repair process of bone. To test this hypothesis, the periosteal cells were digested and collected for flow cytometry analysis with or without bone injury (Fig. 3A). The flow cytometry analysis revealed that the total macrophage population and the pro-inflammatory M1 macrophage population were significantly decreased in the *Prx1*^*Cre*^*;Tnc*^*fl/fl*^ mice 2 days after the bone injury, but not in the uninjured bone (Figs. 3B, C and S3A, B). The expression of pro-inflammation markers including *IL-1b*, *IL-6* and *Nos2* were reduced in macrophages sorted from *Prx1*^*Cre*^*;Tnc*^*fl/fl*^ mice compared with that sorted from the control mice (Fig. S3C). In line with the decreased macrophage recruitment, leukocyte migration and chemotaxis related genes were down-regulated in tdTomato+ cells isolated from *Prx1*^*Cre*^*;Tnc*^*fl/fl*^*;Ai9/+* mice 2 days after bone injury (Figure S4A–C). The immunofluorescence staining and quantification of the periosteal macrophages by CD68 in the uninjured and injured *Prx1*^*Cre*^*;Ai9/+* and *Prx1*^*Cre*^*;Tnc*^*fl/fl*^*;Ai9/+* mice showed that the increase of CD68+ macrophages at day 2 post injury was attenuated in the *Prx1*^*Cre*^*;Tnc*^*fl/fl*^ mice. The shortest distance between *Prx1*+ cells and macrophages at day 2 post injury decreased in *Prx1*^*Cre*^*;Ai9/+* but not *Prx1*^*Cre*^*;Tnc*^*fl/fl*^*;Ai9/+* mice, implying that the recruitment of macrophages by *Prx1*+ cells was impaired by TNC deficiency (Fig. 3D–F). We utilized the transwell assay to examine if TNC was required by *Prx1*+ cells for macrophage recruitment in vitro. In the assay, periosteal *Prx1*+ cells from *Prx1*^*Cre*^*;Ai9/+* or *Prx1*^*Cre*^*;Tnc*^*fl/fl*^*;Ai9/+* mice were seeded in the lower chamber while the WT periosteal macrophages from injured periosteum were seeded in the upper chamber, where the serum-free media forced the macrophages to migrate toward the lower chamber (Fig. 3J). The assay showed that the migration velocity of macrophages was repressed in the *Tnc*-deficient periosteal progenitor cells, compared with WT periosteal progenitor cells (Fig. 3K, L). In the cell adhesion assay, the macrophages from injured periosteum stained by calcein-AM were seeded on the confluent periosteal *Prx1*+ cells from injured *Prx1*^*Cre*^*;Ai9/+* or *Prx1*^*Cre*^*;Tnc*^*fl/fl*^*;Ai9/+* for 30 min before the nonadherent cells were washed out (Fig. 3G). The deficiency of *Tnc* in the periosteal cells reduced the adhesion of macrophages (Fig. 3H, I).

Since we observed that the recruitment of macrophages after bone injury in *Prx1*^*Cre*^*;Tnc*^*fl/fl*^ mice was damaged, we hypothesized that the increased macrophages after bone injury were necessary for efficient bone repair. To test that hypothesis, we examined the bone regeneration in *Tnc*^*fl/fl*^ mice and *Prx1*^*Cre*^*;Tnc*^*fl/fl*^ mice after the implantation of macrophages sorted from the periosteum of injured bone. The repaired bones were harvested for μ-CT analysis 2 weeks after the bone injury and the implantation of macrophages. The regeneration efficiency significantly increased in the *Prx1*^*Cre*^*;Tnc*^*fl/fl*^ mice by the implanted macrophages (Fig. 3M–O). On the other hand, to investigate if macrophages are required by the enhancement of bone regeneration by TNC, we depleted the periosteal macrophages in the early stage of bone repair by clodronate liposomes (Fig. S3D, E). The micro-CT quantification showed that depletion of periosteal macrophages decreased bone repair and TNC delivery could not promote bone regeneration without periosteal macrophages (Figure S3F, G). Taken together, the data above suggested that TNC was essential for the recruitment of macrophages by *Prx1*+ cells during bone repair and that enough inflammatory macrophages were necessary for efficient bone regeneration.

### TNC promoted the recruitment of macrophages through ITGA7

**Fig. 4:**
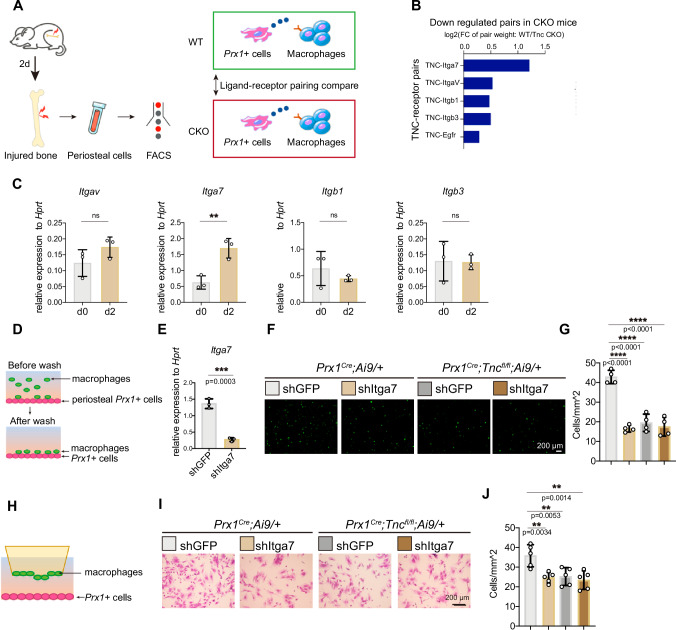
TNC promoted the recruitment of macrophage through ITGA7. **A** Scheme for experiment and analysis of cell crosstalk mediated by *Prx1*+ cells-derived TNC and the receptor of TNC. 2 days after the femoral bone of *Prx1*^*Cre*^*;Ai9/+* and *Prx1*^*Cre*^*;Tnc*^*fl/fl*^ *;Ai9/+* mice were drill injured, periosteal cells were isolated for the FACS of *Prx1*+ cells, labeling by tdTomato, and macrophages for RNA-seq. With the RNA-seq data of the two types of cells, NATMI identified the pairs of TNC and its receptors, which were ranked by their weights calculated by the expression foldchange between control and CKO group. **B** Summary of the analysis result described in (**A**). **C** RT-qPCR examination of the gene expression of the indicated integrins in the periosteal macrophages isolated by FACS at d0 and d2. *n* = 3. **D** Experiment scheme of the adhesion assay: confluent primary periosteal *Prx1*+ cells from the periosteum of mice at d2 were seeded in the culture dish and the macrophages from the periosteum of mice at d2 treated with indicated lentivirus and then stained by Calcein-AM were added in the dish for 30 min for cell adhesion before the un-adhered cells were washed. **E** Itga7 gene expression examined by RT-qPCR in macrophages infected with indicated lentivirus. *n* = 3. Representative images (**F**) and quantification (**G**) of the adhered macrophages in the adhesion assay. *n* = 4. **H** Experiment scheme of the trans-well assay: the periosteal *Prx1*+ cells from the periosteum of mice at d2 were seeded in the lower chamber to induce the migration of the indicated macrophages from the periosteum of mice at d2 seeded in the upper chamber. Representative images (**I**) and quantification (**J**) of the migrated macrophages (stained by crystal violet). *n* = 5. The statistical significance of differences was assessed using two-tailed Student’s unpaired t test or one-way ANOVA. All bar graphs are presented as the mean ± SD.

To further explore what mediated the recruitment of macrophages by periosteal *Prx1*+ cells-derived TNC, we sorted the tdTomato+ cells and macrophages from the periosteum of *Prx1*^*Cre*^*;Tnc*^*fl/fl*^*;Ai9/+* and *Prx1*^*Cre*^*;Ai9/+* mice 2 days after the bone injury and conducted the cell communication analysis with the transcriptome data of these cells through utilizing the analysis tool NATMI (Network Analysis Toolkit for Multicellular Interactions) (Fig. 4A). The score of ligand-receptor pairs in the two kinds of periosteal cells from WT and TNC-deficient mice were compared to find the changed ligand-receptor interaction in the absence of TNC. The cell communication analysis revealed several integrins as possible receptors of TNC during the early stage of bone repair. Among the pairs that enriched in the *Tnc*-deficient mice compared with the control mice, the TNC-ITGA7 (Integrin α7) pair ranked highest. (Fig. 4B). In addition, the expression foldchange of *Itga7* in the periosteal macrophages after bone injury ranked the first among the candidates (Fig. 4C), which suggested that *Itga7* in macrophages was upregulated for responding to bone injury.

To test how *Itga7* affects the crosstalk between periosteal macrophages and periosteal *Prx1*+ cells, we next performed the cell adhesion assay and transwell migration assay between periosteal *Prx1*+ cells and macrophages treated with control or shItga7 lentivirus. The macrophages were stained by Calcein-AM and seeded in the 24-well plate with a confluent monolayer of periosteal *Prx1*+ cells to allow the macrophages to adhere to the periosteal *Prx1*+ cells. After 30 min of adhesion, the cells that did not adhere were removed. Then the adherent macrophages were counted for statistical analysis (Fig. 4D). The shRNA lentivirus successfully decreased the expression of *Itga7* indicated by the RT-qPCR examination (Fig. 4E). The knock-down of *Itga7* in macrophages impaired the adhesion of macrophages to the WT periosteal progenitor cells but not the TNC-deficient periosteal progenitor cells (Fig. 4F, G). In the transwell migration assay, macrophages infected by shItga7 or control lentivirus were seeded in the upper chamber of the coculture system, while the periosteal *Prx1*+ cells were seeded in the lower chamber to induce the migration of the macrophages (Fig. 4H). Knock-down of *Itga7* reduced the migration velocity of the macrophages in the upper chamber to the WT periosteal progenitor cells but not the TNC-deficient periosteal progenitor cells (Fig. 4I, J). The data above implied that periosteal *Prx1*+ cell-derived TNC mediated the adhesion and migration of macrophages through ITGA7 during bone repair.

### mSSCs decreased in periosteum of *Prx1*^*Cre*^*;Tnc*^*fl/fl*^ mice after bone injury

**Fig. 5:**
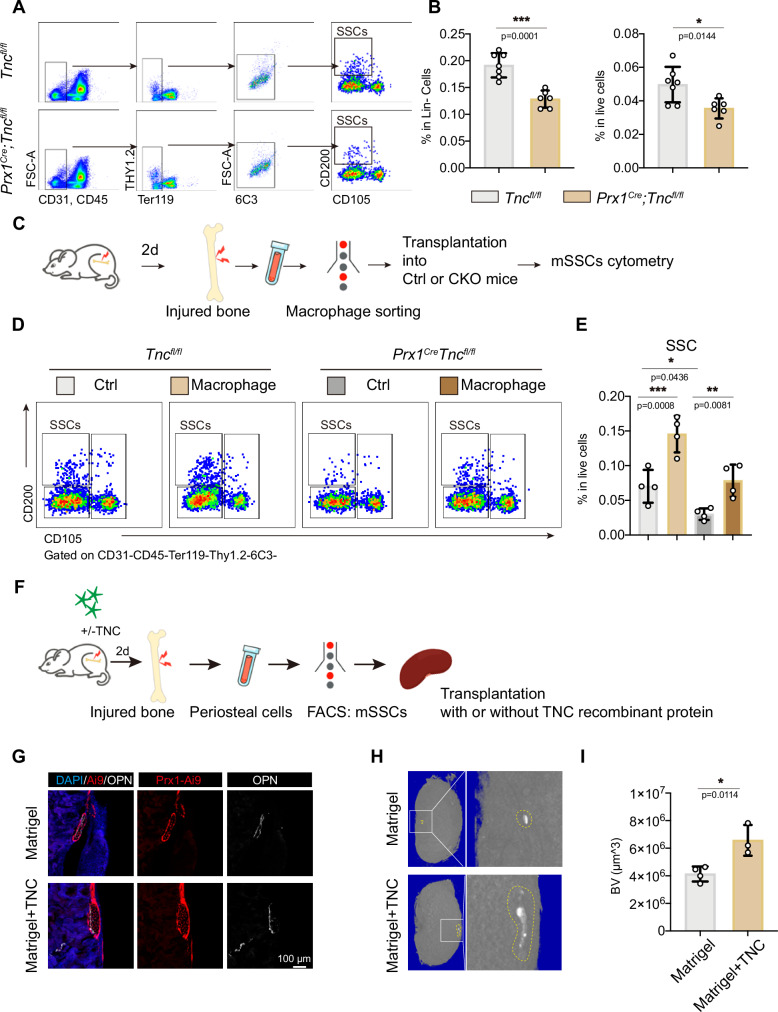
mSSCs decreased in *Prx1*^*Cre*^;*Tnc*^*fl/fl*^ mice after bone injury. **A** Flow cytometry analysis of mSSCs in the periosteum at 2-day post injury. (**B**) Quantification of the percentage of mSSCs in the periosteal cells at 2-day post injury. *n* = 7 for Ctrl, *n* = 6 for CKO. (**C**) Experiment scheme: 2 days after bone drill injury of WT mice, macrophages of periosteum were sorted for transplantation to drill-injured *Tnc*^*fl/fl*^ and *Prx1*^*Cre*^;*Tnc*^*fl/fl*^ mice. 2 days after the transplantation of macrophages, the percentage of mSSCs in periosteum was analyzed by flow cytometry. **D** Flow cytometry analysis of the periosteal mSSCs at 2-day post injury and macrophages transplantation. **E** Quantification of the percentage of mSSCs in the periosteal cells at 2-day post injury. *n* = 4. **F** Experiment scheme: femoral bone of *Prx1*^*Cre*^;Ai9/+ mice were drill injured with or without the treatment of TNC. 2 days after the injury, mSSCs in the periosteum were sorted for the transplantation to renal capsule. **G** Representative immunofluorescence images of the kidneys with mSSCs transplantation, where the transplanted cells were labeled by tdTomato and bone was labeled by OPN. Representative micro-CT images (**H**) of the kidneys with indicated treatment where the yellow dashed line illustrating the new-formed bone in the renal capsule and the quantification (**I**) of bone volume per tissue volume (BV/TV) of the newly formed bone in the renal capsule. *n* = 4 for Ctrl, *n* = 3 for treatment group. The statistical significance of differences was assessed using two-tailed Student’s unpaired t test or two-way ANOVA. All bar graphs are presented as the mean ± SD.

Mouse skeletal stem cells (mSSCs) are responsible for bone development and injury repair [20]. The activation and proliferation of mSSCs are crucial for successful bone repair [21]. Since the immune microenvironment is crucial for stem cell maintenance and activation, we hypothesized that the pro-inflammatory microenvironment of the periosteum after the bone injury is essential for repair-promoting regulation of mSSC population. Flow cytometry analysis revealed that the ratio of mSSCs in the periosteum after bone injury was reduced by TNC deficiency (Fig. 5A, B). While the ratio of mSSCs in periosteum showed no difference between *Prx1*^*Cre*^*;Tnc*^*fl/fl*^ mice and control mice without bone injury (Fig. S5A, B). To demonstrate the positive influence of pro-inflammatory macrophages on mSSCs during the early stage of bone injury repair, macrophages sorted from injured bone of WT mice were implanted into the injured site of *Prx1*^*Cre*^*;Tnc*^*fl/fl*^ mice and their littermate control (Fig. 5C). The periosteal cells were digested for cytometry and analysis of mSSCs ratio 2 days after the drill injury and the implantation of macrophages. The cytometry analysis showed that macrophage implantation could rescue the decreased mSSC ratio in *Prx1*^*Cre*^*;Tnc*^*fl/fl*^ mice (Fig. 5D, E). Taken together, these data suggested that the pro-inflammatory macrophages acted as a niche which was beneficial to the stimulation of mSSCs during bone repair, whereas the upregulated TNC after bone injury was necessary for the maintenance of this pro-inflammatory microenvironment to ensure efficient bone regeneration.

Furthermore, we designed the transplantation experiments to examine if TNC protein and the increased pro-inflammatory macrophages could enhance the bone formation ability of mSSCs in the injured periosteum. In this experiment, *Prx1*^*Cre*^*;Ai9/+* mice were drill injured with or without TNC delivery on the injury sites. 2 days after the injury, tdTomato+ SSCs sorted from the injured *Prx1*^*Cre*^*;Ai9/+* mice were transplanted into the renal capsule of the WT recipient mice (Fig. 5F). 2 months after the transplantation, the kidneys of the recipient mice were harvested for bone formation analysis. μ-CT scanning quantification and immunofluorescence staining showed that the addition of TNC enhanced the bone formation of periosteal mSSCs in the renal capsule transplantation (Fig. 5G–I). The results above demonstrated that local delivery of TNC enhanced the osteogenic capacity of mSSCs.

### Local delivery of TNC improved bone repair after aging

**Fig. 6:**
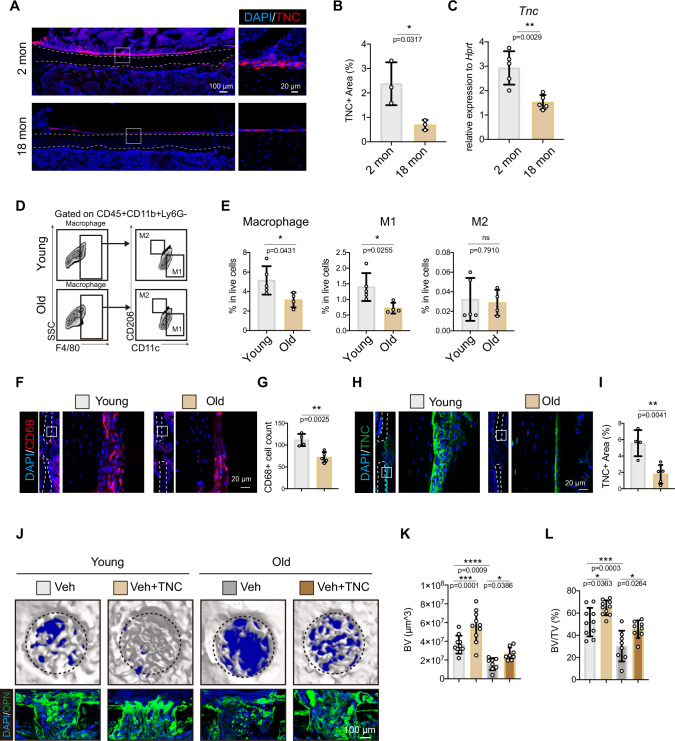
Local delivery of TNC improved bone regeneration after aging. Representative immunofluorescence images of TNC in the homeostatic periosteum (**A**) and the quantification of TNC area in young and old mice (**B**). **C** Quantitative RT-qPCR detection of the *Tnc* gene expression in periosteum of young and old mice. Flow cytometry of macrophages at d2 of drill injury (**D**) and quantification results (**E**) in periosteum of young and old mice. *n* = 5 for young group, *n* = 4 for old group. (**F,** **G**) Representative immunofluorescence images of macrophages (F) and quantification (G) in the periosteum of young and old mice. Representative immunofluorescence images of TNC (**H**) and quantification of TNC area (**I**) in the periosteum of young and old mice 2 days after drill injury. n = 4 for young group, *n* = 5 for old group. **J** Representative micro-CT images and immunofluorescence images of the drill holes in the femoral bone of WT young and old mice at 14-day post bone defect injury, where the black dashed circle illustrated the regenerated bone in the drill holes. Quantitative measurements of bone volume (BV) (**K**) and bone volume per tissue volume (BV/TV) of the newly formed bone in the drill holes (**L**). *n* = 8 or 10. The statistical significance of differences was assessed using two-tailed Student’s unpaired *t* test or two-way ANOVA. All bar graphs are presented as the mean ± SD.

Aging is associated with dysregulated immune microenvironment in bone [22]. The comparison between young and old mice without bone injury found that the expression of TNC in the periosteum decreased significantly with aging (Fig. 6A–C). Aging also decreased periosteal macrophages 2 days after bone defect injury (Fig. 6D–G). The upregulation of TNC after bone injury was impeded in old mice (Fig. 6H, I).

It has been demonstrated that TNC is required by *Prx1*+ cells for efficient bone regeneration. To test if exogenous TNC can promote the bone regeneration process through optimizing immune response in aged mice, we delivered the Matrigel with or without the TNC protein to the drilled hole in the femurs. The cytometry analysis showed that exogenous TNC protein increased the ratio of macrophages and mSSCs in the periosteum during the early stage of bone injury repair (Fig. S6A–D). The μ-CT and the quantification results showed that the delivery of TNC accelerated the bone regeneration in both young and old mice (Fig. 6J–L). These results showed that the decreased TNC in homeostatic periosteum and impeded upregulation of TNC in injured bone with aging may contribute to the significantly decreased bone repair capacity of aged bone and the supplement of TNC enhanced bone regeneration in both young and old mice.

## Discussion

In the present study, we proposed that the periosteal ECM TNC is crucial for efficient bone repair by regulating the interaction between immune cells and periosteal skeletal stem cells. *Prx1*+ lineage cells-derived TNC impels the recruitment of macrophages in the early stage of bone repair, maintaining a repair-promoting niche for the mSSCs and leading to enhanced bone formation. Local delivery of TNC is sufficient to improve the immune microenvironment and thereby augment the bone formation ability of mSSCs.

As a thin layer of connective tissue encompassing most part of the long bones, the periosteum contains abundant cell source and ECM enabling rapid response and remarkable regenerative ability after the bone injury [23, 24].

Skeletal stem/progenitor cells have been found in different anatomical sites including the periosteum and bone marrow [25, 26]. Previous study has shown that periosteal cells and bone marrow stromal/stem cells (BMSCs) may be derived from a common embryonic mesenchymal lineage [27]. Both types of cells participate in bone regeneration after bone injury [28–30]. Lineage tracing and flow cytometry revealed that the periosteal cells and BMSCs shared some of the cell surface markers like Hox11, PDGFRβ [31–33]. Despite the shared markers, it has been shown that the postnatal periosteal cells exhibit greater clonogenicity and differentiation capacity than BMSCs both in vitro and ex vivo [27]. Given the complexity of the skeletal stem/progenitor cells composition, it still remains unclear whether these lineages are interdependent [34]. Besides, more specific markers for skeletal stem/progenitor in periosteum and bone marrow and the relationship between these populations under steady and injury conditions may be proposed in the future.

ECM, including Periostin in the periosteum, is an important factor that distinguishes the cells from the periosteum and bone marrow in terms of regeneration capacity [27]. Here in the present study, we demonstrated that the periosteum-derived ECM TNC was required by bone defect repair via providing a sustainable repair-promoting microenvironment in the early stage of bone regeneration, which deepened our understanding of the function of ECM in the periosteum.

Although it has been reported that the knockout of *Tnc* in the whole body of mice did not influence the normal development and breeding of the mice, TNC was found to be important in the repair process of multiple tissues [18, 19]. During embryogenesis, TNC appears at many morphogenesis sites, such as limb buds, which makes it a marker of perichondrium, periosteum, ligaments, and tendons [14]. TNC is also dynamically expressed around budding epithelia in developing epithelial organs including kidney, lung, and mammary glands [35]. During adulthood, TNC is steadily expressed in the periosteum of long bones. After the bone defect, the expression of TNC in the periosteum increases promptly. This characteristic upregulation of this ECM happens in various pathological situations including tissue trauma and cancer. It is intriguing to explore the upstreaming signal that triggers the upregulation of TNC when abnormalities invade the tissues and the tissue specificity of these upstreaming signals. A study about glioma brain tumor found that the hypoxia microenvironment in the tumor and the key transcription factor hypoxia-inducible factor 1 alpha (HIF1α) led to the upregulation of TNC through binding directly to the promotor of *Tnc* [36].

Derived from monocytes or residing in the tissue locally, the macrophage is one of the cell types that are recruited to the injury site at the earliest stage. This cell population plays a versatile role in tissue repair and regeneration because of its heterogeneous compositions and high plasticity. Recently, the crosstalk between macrophages and the tissue-resident cells to promote regeneration has been revealed in many tissues. Interaction between neurons and macrophages has been reported in the injury repair of skin tissue, where sensory nerve had the potential to sustain the tissue repair function of macrophages via the release of specific signal molecules [8, 37].

Proteins and metabolites from macrophages have been demonstrated to direct the regulation of tissue stem cells by macrophages during injury repair. In skeletal muscle, via the receptor C-C motif chemokine receptor type 5 (Ccr5) on the muscle stem cells, the cytokine nicotinamide phosphoribosyltransferase (NAMPT) secreted by macrophage after injury formed a transient stem-cell-activating niche to provide the resident stem cells with proliferating-inducing cues [38]. In the aspect of metabolism, macrophage-derived glutamine was transported to muscle stem cells by the glutamine transporter SLC1A5 and promoted the proliferation and differentiation of muscle stem cells [39]. In bone repair, factors secreted by macrophages like low-density lipoprotein receptor-related protein 1 (*Lrp1*) was found to improve fracture healing through promoting osteoblast differentiation of bone marrow stromal cells [40]. The influence of the macrophage-derived factors on bone repair has been widely examined through in vivo and in vitro research, which demonstrates the osteoblast-differentiation-promoting function of macrophages [41, 42]. During bone injury repair, however, how the macrophages fit in with the skeletal regenerative context and cooperate with other cell types to sustain efficient bone regeneration still remains to be investigated. Herein, we focused on the recruitment of macrophages by periosteal *Prx1*+ lineage cells and revealed the regeneration-promoting niche the macrophages contributed to the mSSCs in the early stage of bone repair.

Advancements in the technology of lineage tracing and single-cell multi-omics analysis further uncovered the essential role of periosteum and the regenerative stem cell populations source in it during bone repair. Following the identification of the mSSCs, the function of mSSCs and the communication between them and other cell types in bone have been discussed. In mandibular defects, the bone repair is driven by mSSCs that depend on the paracrine signals from Schwann cells [21]. It was proposed that aged SSC lineage drives hematopoietic aging through promoting osteoclastic activity and myeloid skewing, which presented dynamic interaction between SSCs and other cell types [43]. Given the essential role of skeletal stem/progenitor cells in homeostasis maintenance and injury repair of bone and the complex cell composition in bone, how these cells receive the signals from their surrounding cells is well worth for further elucidation. In the present study, we proposed the role of pro-inflammatory macrophages, that are recruited by the periosteal ECM TNC, as a repair-promoting niche which enhances bone injury repair and regeneration. Furthermore, as a stable ECM protein, TNC may be useful in the application for bone repair accelerating clinically.

## Materials and methods

### Data analysis

The immunofluorescence analysis was performed by using FIJI and Imaris. The data were presented as mean ± SD. All experiments were repeated at least three times and the representative images were presented eventually. RNA-seq data has been uploaded in figshare.com, 10.6084/m9.figshare.27328593.v1.

### Mice

*Tnc* flox mice were purchased from GemPharmatech Co. Ltd. *Prx1*-Cre (stock no: 005584) mice were obtained from the Jackson Laboratory and were maintained in the laboratory for more than 10 generations. Ai9 reporter mice were provided by Zilong Qiu (Chinese Academy of Sciences, Shanghai, China). All mouse experiments compiled with all relevant ethical regulations and were performed according to protocols approved by the Animal Care and Use Committee of the Shanghai Institute of Biochemistry and Cell Biology, Chinese Academy of Sciences (protocol number: SIBCB-S350-2312-42).

### Micro-CT scan and analysis

CT scanning of samples was performed by skyscan 1272 (Bruker) at a 9 μm resolution. For the samples with bone defects, 50 slides of the defect regions were selected for quantitative analysis of bone volume (BV) and bone volume per tissue volume (BV/TV) of regenerated bone. Three-dimensional reconstructions were created by stacking the two-dimensional images from the indicated regions with CTVox.

### Bone injury

The drill hole injury in the bone defect model was realized by an electric drill with a diameter of 0.5 mm. Hair on the skin of femoral bone of the anaesthetized mouse was removed. After disinfection with ethanol, an approximately 8-mm longitudinal skin incision was made above the tibia. Exposing the anterior side of bone surface through splitting the muscle covered on it. A drill hole was made on the anterior portion of the tibia by the electric drill. The incised muscle and skin were closed with silk sutures. For local delivery of TNC (Merck, cc065), the protein was mixed with Matrigel in the concentration of 500 ng/μl and added in the injured sites before the suture of the muscle and skin. For the transplantation of the periosteal macrophage, periosteal cells of the drill-injured bone were digested from femurs with bone marrow flushed thoroughly. The sorted cells were mixed with Matrigel and added in the injured sites before the suture of the muscle and skin.

### Osteoblast differentiation

For osteoblast differentiation, cells were induced by α-MEM with 10% FBS, 1% penicillin/streptomycin, 50 μg/ml L-ascorbic acid (Sigma, A5960), and 1.08 mg/ml β-Glycerophosphate disodium salt hydrate (Sigma, G9422). For ALP staining, cells were fixed with 4% paraformaldehyde for 10 min on day 7 and then stained according to the manual of BCIP/NBT Alkaline Phosphatase Color Development Kit (Beyotime, C3206). Bone nodule formation was stained with 1 mg/ml alizarin red s solution (pH 5.5) after 21 days of induction.

### Adhesion assay and migration assay

For adhesion assay, macrophages stained by calcein-AM in room temperature for 30 min and wash were added to culture dish with confluent cells. After 30 min for adhesion, the nonadherent macrophages were washed out by PBS for three times. For trans-well migration assay, the trans-well occulture system (Corning, Transwell) was used. *Prx1*+ cells were seeded in the lower chamber with full medium. Macrophages were seeded on the upper chamber in media without serum so the macrophages can be induced to migrate to the down side of bottom of the upper chamber. After 48 h, cells in the upper chamber were fixed with 4% PFA and the cells on the up side were removed by cotton swabs. Then the migrated cells on the down side were stained by crystal violet.

### Flow cytometry analysis

Periosteal cells were extracted from femurs of injured or uninjured mice. Bone marrow was flushed thoroughly. Then the cut-up bone pieces were digested by α-MEM with 1 mg/ml collagenase (Sigma, C0130), 2 mg/ml Dispase II (Sigma, D4693) and 1U/ml DNase I (sigma, DN25) for 10 min. Then the digested cells were discarded. Then the bones were digested for another three times for 15 min. The digested cells were collected into a 15 ml centrifuge tube containing α-MEM (Corning) supplemented with 10% fetal bovine serum (FBS) and centrifuged at 1200 g for 5 min. The cell pellet was re-suspended with PBS with 2% FBS. The red blood cells were removed by RBC lysis buffer for 5 min (Beyotime, C3702). Wash the cells twice by centrifugation at 1200 g for 5 min with ice-cold PBS with 2% FBS. The cells were stained with PerCP/Cy5.5 anti-CD31 (BioLegend, 102522), PerCP/Cy5.5 anti-CD45 (BioLegend, 103132), PerCP/Cy5.5 anti-mouse TER-119 (BioLegend, 116228), PerCP/Cy5.5 anti-mouse 6C3/Ly-51 (BioLegend, 108316), Brilliant Violet 605 anti-mouse CD90.2 (BioLegend, 140317), PE/Cy7 anti-mouse CD105 (BioLegend, 120409), APC anti-mouse CD200 (BioLegend, 123809) FITC anti-mouse Ly6G (BioLegend, 127605), BV605 anti-mouse F4/80 (BioLegend, 123133), APC anti-mouse CD11b (BioLegend, 101212), APC/Cy7 anti-mouse CD11b (BioLegend, 101226), PE anti-mouse CD206 (BioLegend, 141706) for 30 min. Then cells were measured by BC Cytoflex LX after twice of wash by centrifugation at 600 g for 5 min with PBS with 2% FBS. The data were then analyzed with the CytExpert software.

### Histology analysis

For skeletal whole-mount staining, mice were eviscerated, and the skin was removed. The resulting samples were incubated in acetone for 48 h after overnight fixation in 95% ethanol. Skeletons were then stained in Alcian blue and Alizarin red solution. Specimens were stored in 1% KOH until the tissue had completely cleared.

### Real-time RT-qPCR analysis

Total RNA was prepared using TRIzol (T9424, Sigma) and was reverse transcribed into cDNA with the PrimeScript RT Reagent Kit (PR037A, TakaRa). The real-time reverse transcriptase RT-qPCR reaction was performed with the BioRad CFX96 system. The primer sets used in this paper are listed in Table S1.

### Macrophages depletion by clodronate liposomes

For the depletion of periosteal macrophages in the early stage of bone repair, 1 day before bone injury and the next 2 days after bone injury, mice in the treatment group were injected with clodronate liposomes through intravenous injection (5 μl/g) and local injection at the injured tibia (50 μl) once a day. While the control group was injected with the same volume of control liposomes.

### Immunofluorescence staining

The dissected bones were fixed in 4% paraformaldehyde for 48 h and incubated in 15% DEPC-EDTA (pH 7.8) for decalcification. The specimens were then embedded for cryosection. Sections were blocked in PBS with 10% horse serum for 1 h and then incubated overnight with corresponding antibodies. The primary antibodies included goat anti-OPN (R&D, AF808, 1:200), rabbit anti-TNC (Abcam, ab-108930, 1:200), mouse anti-CD68 (Biolegend, a37008, 1:200). DAPI (sigma, D8417) was used for counterstaining. Slides were mounted with fluorescence mounting medium (Dako, S3023).

### Statistics

Statistical analysis was performed using GraphPad Prism 8 software (GraphPad Software). Cell-based experiments were performed at least twice. Animals were randomized into different groups and at least three mice were used for each group unless otherwise stated. The data were presented as mean ± s.d. Two-tailed Student’s *t* test was used to compare the effects of the two groups. One-way ANOVA or two-way ANOVA were used for analysis of three or more groups.

## Data and materials availability

All data and genetic material used for this paper are available from the authors on request.

## Supplementary information

Tenascin-C promotes bone regeneration via inflammatory macrophages

Supplementary Table 1
