# Supplementary material for: Tenascin-C promotes bone regeneration via inflammatory macrophages
Source: Cell Death Differ. 2025 Jan 10;32(4):763–75. doi: 10.1038/s41418-024-01429-9 (PMC11982535; doi:10.1038/s41418-024-01429-9)
Supplement: Supplementary file 1 — Tenascin-C promotes bone regeneration via inflammatory macrophages [file 41418_2024_1429_MOESM1_ESM.pdf]

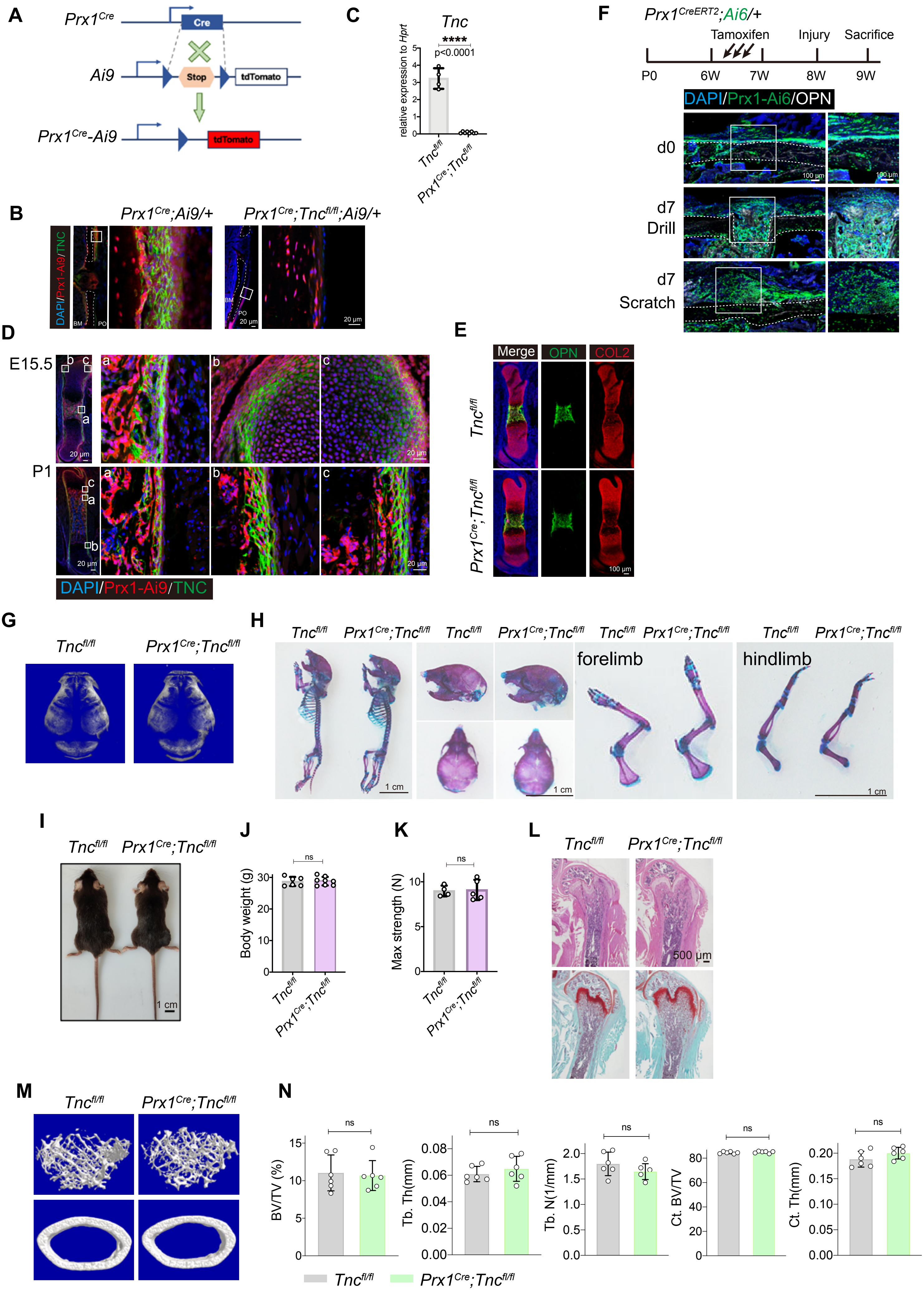
A
Prx1Cre
Cre
Ai9
Stop
tdTomato
Prx1Cre-Ai9
tdTomato
B
Prx1Cre;Ai9/+
Prx1Cre;Tncfl/fl;Ai9/+
DAPI/Prx1-Ai9/TNC
BM
PO
20 μm
C
Tnc
****
p<0.0001
relative expression to Hprt
Tncfl/fl
Prx1Cre;Tncfl/fl
D
E15.5
P1
DAPI/Prx1-Ai9/TNC
20 μm
E
Merge
OPN
COL2
Tncfl/fl
Prx1Cre;Tncfl/fl
100 μm
F
Prx1CreERT2;Ai6/+
Tamoxifen
Injury
Sacrifice
P0
6W
7W
8W
9W
DAPI/Prx1-Ai6/OPN
d0
d7 Drill
d7 Scratch
100 μm
G
Tncfl/fl
Prx1Cre;Tncfl/fl
H
forelimb
hindlimb
1 cm
I
J
Body weight (g)
ns
K
Max strength (N)
L
500 μm
M
N
BV/TV (%)
Tb. Th(mm)
Tb. N(1/mm)
Ct. BV/TV
Ct. Th(mm)

Fig. S1 Normal bone development of *Prx1*^{Cre}*;Tnc*$^{fl/fl}$ mice. (**A**) Construction strategy of the *Prx1*^{Cre}*;Ai9/+* mice. (**B)** Representative immunofluorescence images showing the expression of TNC in the periosteum of *Prx1*^{Cre}; Ai9/+ and *Prx1*^{Cre};*Tnc*$^{fl/fl}$*;Ai9/+* mice at d2, where the white dashed line illustrated the periosteum of mice and the white square illustrates the amplified part of the periosteum shown on the right side. BM, bone marrow, PO, periosteum. (**C)** Quantitative RT-PCR detection of the *Tnc* gene expression in periosteal cells of *Tnc*$^{fl/fl}$ and *Prx1*^{Cre};*Tnc*$^{fl/fl}$ mice at d2. n=3. (**D, E)** Immunofluorescence images of TNC in femoral bone of *Prx1*^{Cre}; Ai9/+ mice at indicated development time points (D) and the immunostaining of OPN and COL2 in the femoral bone of *Tnc*$^{fl/fl}$ and *Prx1*^{Cre}*;Tnc*$^{fl/fl}$ mice at E15.5 (E). (**F)** Tracing images of the *Prx1*+ cells in uninjured periosteum and periosteum with drill injury and scratch injury at d7. (**G)** Micro-CT images of calvarial bone of *Tnc*$^{fl/fl}$ and *Prx1*^{Cre};*Tnc*$^{fl/fl}$ mice at P5. (**H)** Whole-mount Alizarin Red S/Alcian Blue staining of *Tnc*$^{fl/fl}$ and *Prx1*^{Cre};*Tnc*$^{fl/fl}$ mice at P7. (**I, J)** Representative gross image (I), quantification of bodyweight (J) of *Tnc*$^{fl/fl}$ and *Prx1*^{Cre};*Tnc*$^{fl/fl}$ mice at 16-week-old. n=6 or 8. (**K)** Quantification of the max strength of femoral bone in three-point-bending assay of *Tnc*$^{fl/fl}$ and *Prx1*^{Cre};*Tnc*$^{fl/fl}$ mice at 8-week-old. n=4 or 5. (**L)** Hematoxylin and eosin (H&E) (left) and Safranin O-Fast green staining (right) of Femoral bone sections of *Tnc*$^{fl/fl}$ and *Prx1*^{Cre};*Tnc*$^{fl/fl}$ mice at 8-week-old. (**M, N)** Representative micro-CT images of femoral trabecular bone (upper panel) and cortical bone (lower panel) (M) and micro-CT quantification (N) of femoral bone for bone volume per tissue volume (BV/TV), trabecular thickness (Tb.Th), trabecular number (Tb.N), BV/TV of cortical bone (Ct. BV/TV) and thickness of cortical bone (Ct. Th) of *Tnc*$^{fl/fl}$ and *Prx1*^{Cre};*Tnc*$^{fl/fl}$ mice at 16-week-old. n=6. The statistical significance of differences was assessed using two-tailed Student's unpaired t test. All bar graphs are presented as the mean ± SD.

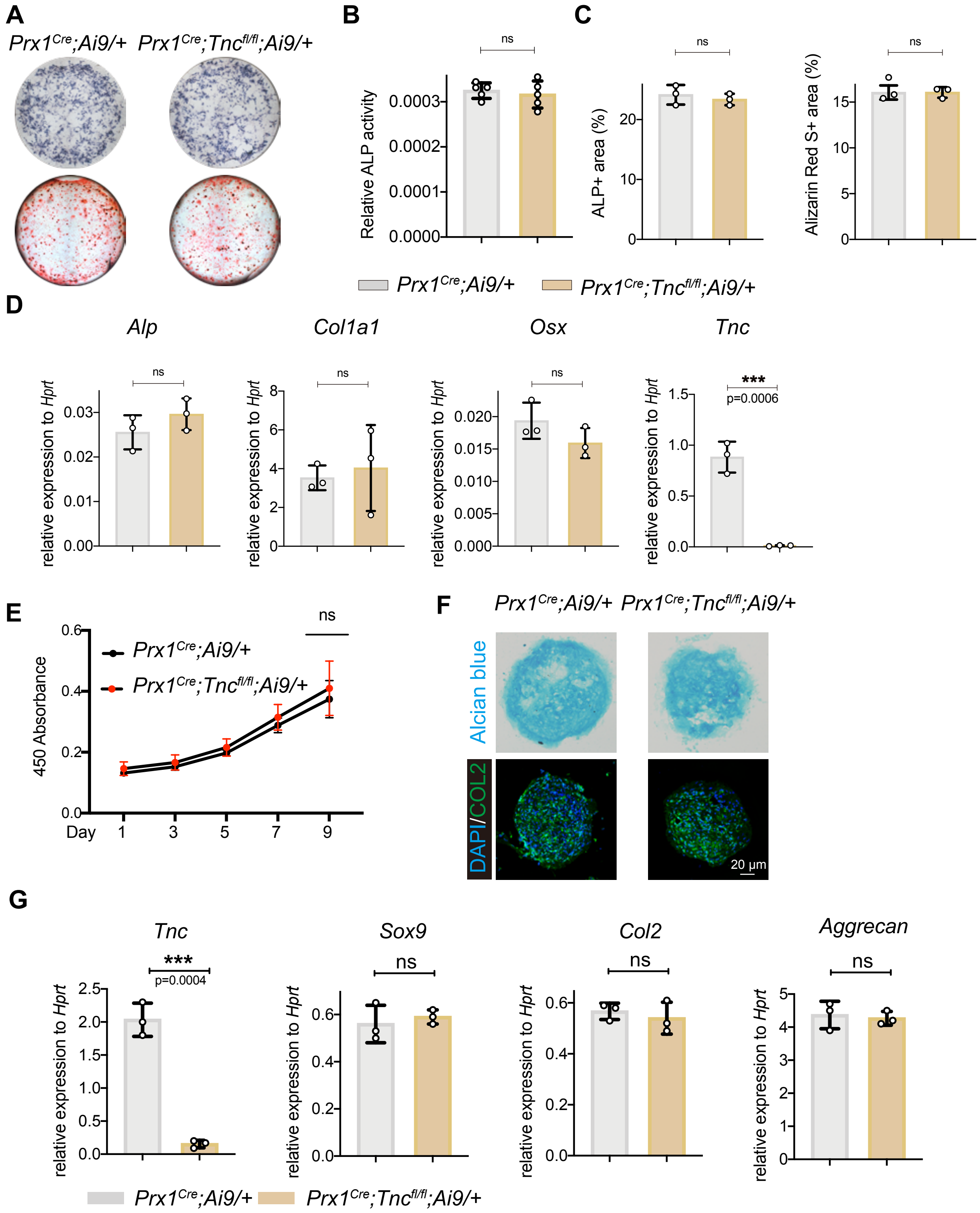

A
Prx1Cre;Ai9/+ Prx1Cre;Tncfl/fl;Ai9/+
B
Relative ALP activity
ns
C
ALP+ area (%)
Alizarin Red S+ area (%)
Prx1Cre;Ai9/+ Prx1Cre;Tncfl/fl;Ai9/+
D
Alp
Col1a1
Osx
Tnc
relative expression to Hprt
***
p=0.0006
E
450 Absorbance
Day
F
Alcian blue
DAPI/COL2
20 μm
G
Tnc
Sox9
Col2
Aggrecan
p=0.0004

Fig. S2 TNC-deficiency in uninjured periosteum did not impact the osteogenic or chondrogenic differentiation of periosteal cells. (**A)** ALP (upper panel) and Alizarin Red S (lower panel) staining of primary *Prx1+* cells from the indicated uninjured mice after induction with osteogenic medium for 7 days. (**B, C)** Relative activity of ALP (B) (n=5) and the quantification of the percentage of Alizarin Red S- and ALP-stained area (C) (n=3). (**D)** Quantitative RT-PCR detection of osteogenic biomarker genes (*Alp, Col1a1, Osx*) and *Tnc* in the osteogenic differentiated cells. n=3. (**E)** Quantification of cell proliferation of the sorted tdTomato+ *Prx1+* periosteal cells from the indicated uninjured mice in cck-8 assay. n=6. (**F, G)** Representative images of the Alcian blue staining and Col2 staining (F) and the expression of the indicated genes of the chondrogenic differentiated periosteal cells after scratch injury (G). n=3. The statistical significance of differences was assessed using two-tailed Student's unpaired t test. All bar graphs are presented as the mean ± SD. The statistical significance of differences was assessed using two-tailed Student's unpaired t test. All bar graphs are presented as the mean ± SD.

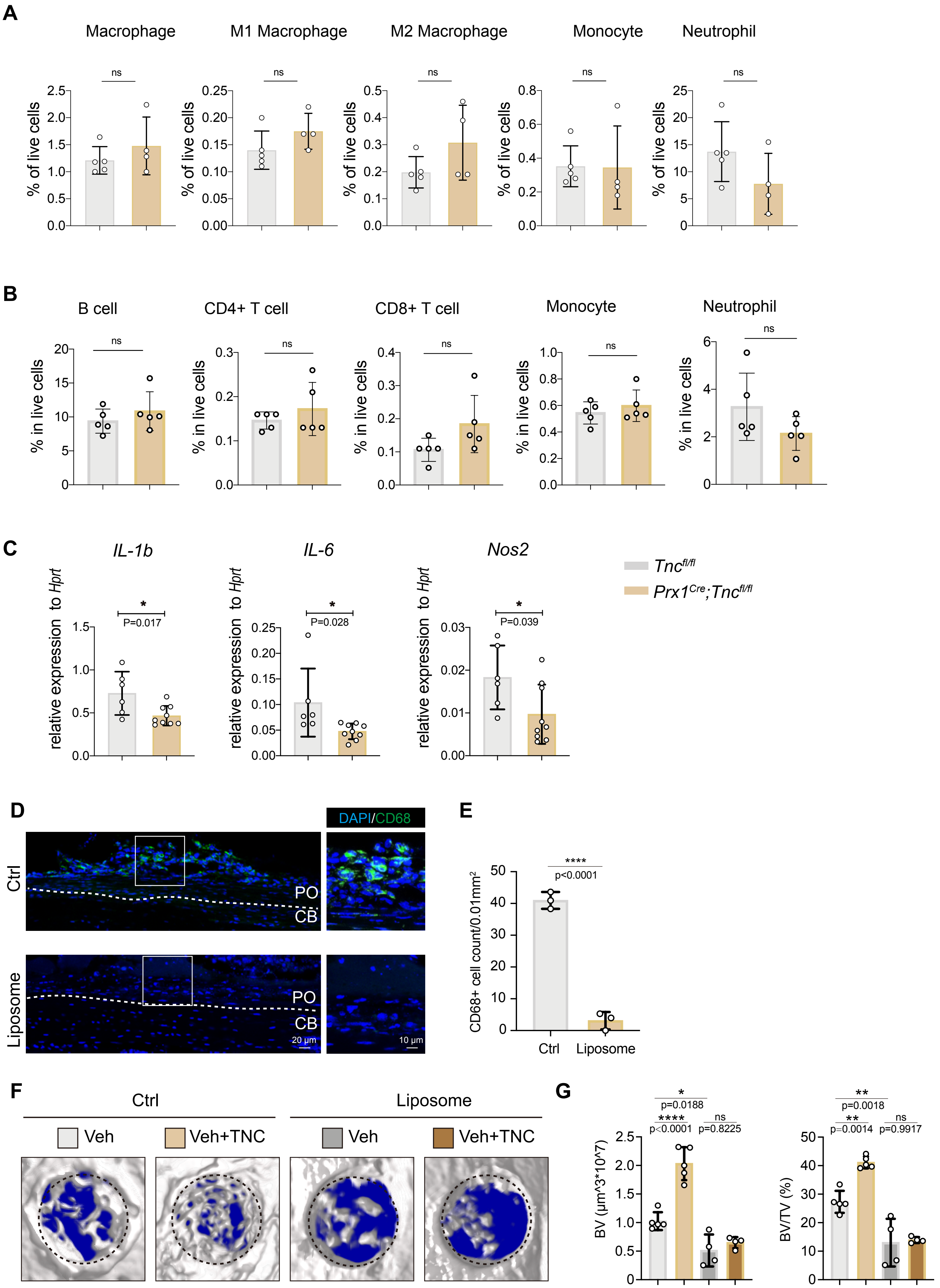
A
Macrophage
M1 Macrophage
M2 Macrophage
Monocyte
Neutrophil
% of live cells
ns
B
B cell
CD4+ T cell
CD8+ T cell
Monocyte
Neutrophil
% in live cells
ns
C
IL-1b
IL-6
Nos2
relative expression to Hprt
P=0.017
P=0.028
P=0.039
Tnc fl/fl
Prx1 Cre;Tnc fl/fl
D
DAPI/CD68
Ctrl
Liposome
PO
CB
20 μm
10 μm
E
CD68+ cell count/0.01mm²
****
p<0.0001
Ctrl
Liposome
F
Ctrl
Liposome
Veh
Veh+TNC
Veh
Veh+TNC
G
BV (μm^3*10^7)
BV/TV (%)
p=0.0188
p<0.0001
p=0.8225
p=0.0018
p=0.0014
p=0.9917
ns

Fig. S3 TNC-deficiency in *Prx1*+ cells did not influence immune microenvironment of periosteum. (**A**) Flow cytometry quantification of the percentage of the indicated immune cells in periosteum of uninjured *Tnc*$^{fl/fl}$ and *Prx1*^{Cre};*Tnc*$^{fl/fl}$ mice. n=4 or 5. (**B)** Flow cytometry quantification of the percentage of the indicated immune cells at 2-day-post injury in the periosteum of *Tnc*$^{fl/fl}$ and *Prx1*^{Cre};*Tnc*$^{fl/fl}$ mice. n=5. (**C)** RT-qPCR quantification of the indicated genes in the sorted macrophages from the periosteum of *Tnc*$^{fl/fl}$ and *Prx1*^{Cre};*Tnc*$^{fl/fl}$ mice at 2-day-posty injury. n=6 or 9. (**D**) Images and (**E**) quantification of periosteal macrophages after treatment of control liposomes or clodronate liposomes. n=3. (**F**) Micro-CT images and (**G**) quantifications of the bone formation after injection of Liposomes and TNC. n=4 or 5. The statistical significance of differences was assessed using two-tailed Student's unpaired t test and two-way ANOVA. All bar graphs are presented as the mean ± SD.

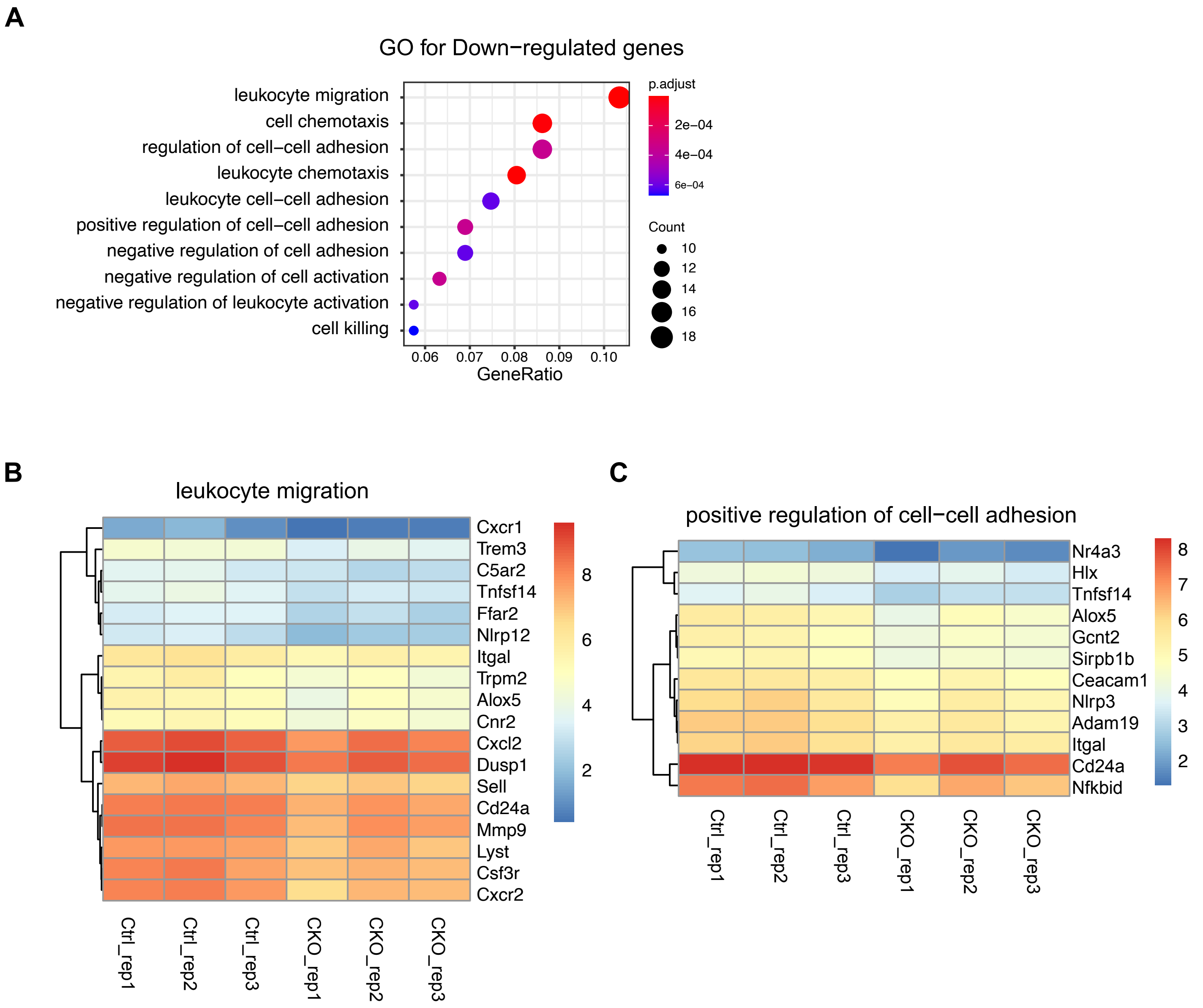

A
GO for Down−regulated genes
leukocyte migration
cell chemotaxis
regulation of cell−cell adhesion
leukocyte chemotaxis
leukocyte cell−cell adhesion
positive regulation of cell−cell adhesion
negative regulation of cell adhesion
negative regulation of cell activation
negative regulation of leukocyte activation
cell killing
0.06
0.07
0.08
0.09
0.10
GeneRatio
p.adjust
2e−04
4e−04
6e−04
Count
10
12
14
16
18
B
leukocyte migration
Cxcr1
Trem3
C5ar2
Tnfsf14
Ffar2
Nlrp12
Itgal
Trpm2
Alox5
Cnr2
Cxcl2
Dusp1
Sell
Cd24a
Mmp9
Lyst
Csf3r
Cxcr2
8
6
4
2
Ctrl_rep1
Ctrl_rep2
Ctrl_rep3
CKO_rep1
CKO_rep2
CKO_rep3
C
positive regulation of cell−cell adhesion
Nr4a3
Hlx
Tnfsf14
Alox5
Gcnt2
Sirpb1b
Ceacam1
Nlrp3
Adam19
Itgal
Cd24a
Nfkbid
8
7
6
5
4
3
2
Ctrl_rep1
Ctrl_rep2
Ctrl_rep3
CKO_rep1
CKO_rep2
CKO_rep3

Fig. S4 Down-regulation of leukocyte migration in TNC-deficient *Prx1*+ cells. **(A)** Gene ontology (GO) analysis of down-regulated genes in tdTomato+ periosteal cells from *Prx1^{Cre};Tnc$^{fl/fl}$;Ai9/*+ mice at 2-day post injury. (**B, C)** Down-regulated genes enriched in the GO of leukocyte migration (B) and positive regulation of cell-cell adhesion (C) in tdTomato+ periosteal cells from *Prx1^{Cre};Tnc$^{fl/fl}$;Ai9/*+ mice at 2-day post injury.

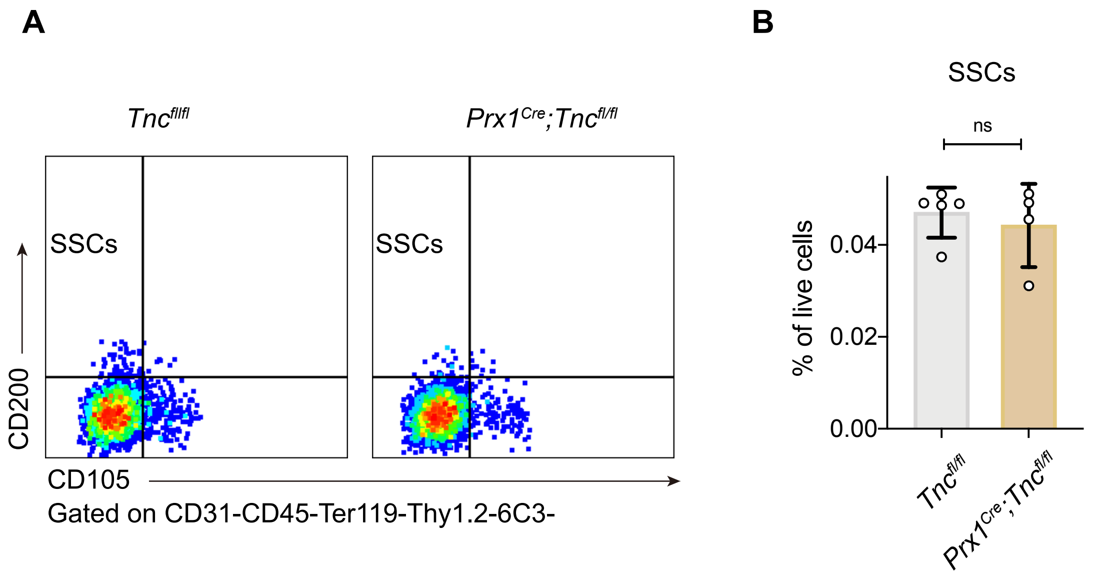
A
Tnc^fl/fl
Prx1^Cre;Tnc^fl/fl
SSCs
SSCs
CD200
CD105
Gated on CD31-CD45-Ter119-Thy1.2-6C3-
B
SSCs
ns
% of live cells
0.04
0.02
0.00
Tnc^fl/fl
Prx1^Cre;Tnc^fl/fl

Fig. S5 TNC-deficiency did not reduce mSSCs in periosteum of uninjured mice. (**A)** Flow cytometry analysis of mSSCs in the periosteum of uninjured mice. (**B)** Quantification of the percentage of mSSCs in the periosteum of uninjured mice. n=4 or 5. The statistical significance of differences was assessed using two-tailed Student's unpaired t test. All bar graphs are presented as the mean ± SD.

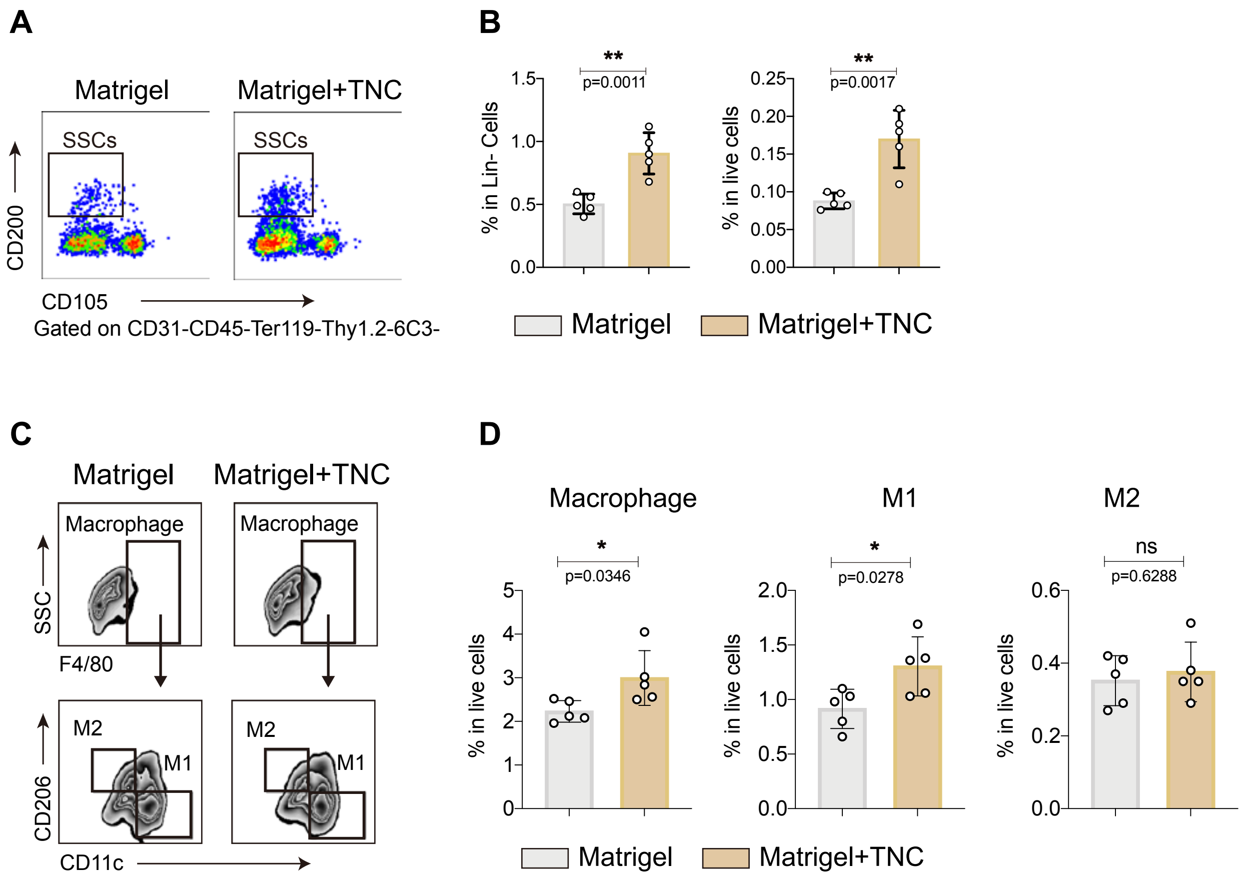

A
Matrigel
Matrigel+TNC
SSCs
SSCs
CD200
CD105
Gated on CD31-CD45-Ter119-Thy1.2-6C3-
B
**
p=0.0011
% in Lin- Cells
**
p=0.0017
% in live cells
Matrigel
Matrigel+TNC
C
Matrigel
Matrigel+TNC
Macrophage
Macrophage
SSC
F4/80
M2
M1
M2
M1
CD206
CD11c
D
Macrophage
M1
M2
*
p=0.0346
*
p=0.0278
ns
p=0.6288
% in live cells
% in live cells
% in live cells
Matrigel
Matrigel+TNC

Fig. S6 TNC delivery in bone defects increased periosteal mSSCs and macrophages. (A, B) Flow cytometry analysis (A) and quantification (B) of the percentage of mSSCs in the periosteum at 2-day post injury and TNC delivery. n=5. **(C, D)** Flow cytometry analysis (C) and quantification (D) of the percentage of total macrophages and M1, M2 macrophages in the periosteal cells of the periosteum at 2-day post injury and TNC delivery. n=5. The statistical significance of differences was assessed using two-tailed Student's unpaired t test. All bar graphs are presented as the mean ± SD.
