# Supplementary material for: Tenascin-C promotes bone regeneration via inflammatory macrophages
Source: Cell Death Differ. 2025 Jan 10;32(4):763–75. doi: 10.1038/s41418-024-01429-9 (PMC11982535; doi:10.1038/s41418-024-01429-9)
Supplement: Supplementary file 2 — Supplementary Table 1 [file 41418_2024_1429_MOESM2_ESM.docx]

Supplementary Table 1. Primer sequences used for real-time quantitative reverse transcriptase polymerase chain reaction (qRT-PCR)

| *Gene* | 5’ to 3’ | | Primers |
| --- | --- | --- | --- |
| *Tnc* | Sense | ACGGCTACCACAGAAGCTG | |
|  | Anti-sense | ATGGCTGTTGTTGCTATGGCA | |
| *Alp* | Sense | CGGGACTGGTACTCGGATAA | |
|  | Anti-sense | ATTCCACGTCGGTTCTGTTC | |
| *Col1a1* | Sense | GCTCCTCTTAGGGGCCACT | |
|  | Anti-sense | CCACGTCTCACCATTGGGG | |
| *Runx2* | Sense | TTTAGGGCGCATTCCTCATC | |
|  | Anti-sense | TGTCCTTGTGGATTAAAAGGACTTG | |
| *Osx* | Sense | CTCTGCTTGAGGAAGAAGCTCAC | |
|  | Anti-sense | CTTCTTTGTGCCTCCTTTCCC | |
| *Itga7* | Sense | GATCGTCCGAGCCAACATCACA | |
|  | Anti-sense | CTAACAGCCCAGCCAGCACT | |
| *Itgb1* | Sense | TTCAGACTTCCGCATTGGCTTTGG | |
|  | Anti-sense | TGGGCTGGTGCAGTTTTGTTCAC | |
| *Itgb3* | Sense | CACGGATGCCAAGACCCATATTG | |
|  | Anti-sense | GTGGAGGCAGAGTAGTGGYYGTC | |
